# Unraveling the Burden of Iron in Neurodegeneration: Intersections with Amyloid Beta Peptide Pathology

**DOI:** 10.1155/2018/2850341

**Published:** 2018-01-31

**Authors:** Romina María Uranga, Gabriela Alejandra Salvador

**Affiliations:** ^1^Instituto de Investigaciones Bioquímicas de Bahía Blanca, Universidad Nacional del Sur (UNS)-Consejo Nacional de Investigaciones Científicas y Técnicas, Bahía Blanca 8000, Argentina; ^2^Departamento de Biología, Bioquímica y Farmacia, UNS, Bahía Blanca 8000, Argentina

## Abstract

Iron overload is a hallmark of many neurodegenerative processes such as Alzheimer's, Parkinson's, and Huntington's diseases. Unbound iron accumulated as a consequence of brain aging is highly reactive with water and oxygen and produces reactive oxygen species (ROS) or free radicals. ROS are toxic compounds able to damage cell membranes, DNA, and mitochondria. Which are the mechanisms involved in neuronal iron homeostasis and in neuronal response to iron-induced oxidative stress constitutes a cutting-edge topic in metalloneurobiology. Increasing our knowledge about the underlying mechanisms that operate in iron accumulation and their consequences would shed light on the comprehension of the molecular events that participate in the pathophysiology of the abovementioned neurodegenerative diseases. In this review, current evidences about iron accumulation in the brain, the signaling mechanisms triggered by metal overload, as well as the interaction between amyloid *β* (A*β*) and iron, will be summarized.

## 1. Introduction

Metals are widely distributed in biological systems and can be referred to as either “biometals” or “toxicological metals,” according to whether they have functional or detrimental roles, respectively, to the organism. In the particular case of transition metals (which are defined as those elements that form at least one ion with a partially filled shell of electrons, e.g., iron, copper, and zinc), when at the appropriate concentration, they participate in the maintenance of normal cellular processes. As with many other substances, the dyshomeostasis of any metal ion, which results in levels outside the normal physiological range, can result in biological damage [1, 2].

Iron is one of the most abundant metals in the Earth's crust [3], and its presence since Earth surface oxygenation (the appearance of oxygen in the atmosphere) has made it possible for life to survive in the oxidative environment. Its high availability, together with its chemical properties, makes it a key component in energy-generating processes [3–6]. This metal, as part of several metalloproteins in the body, is crucial in sensing and transporting oxygen, transferring electrons, and catalyzing many reactions [7]. The biological functions of iron rely upon its chemical properties: it is able to dynamically and flexibly form several coordination complexes with organic ligands, and it has a favorable redox potential to switch between Fe^2+^ (ferrous state) and Fe^3+^ (ferric state). Important to note is the fact that iron bioavailability is limited in aerobic conditions because Fe^2+^ is readily oxidized in solution to Fe^3+^, which is insoluble at a physiological pH [8]. The molecular mechanisms of iron absorption, storage, and homeostasis have been further defined through the discovery of a number of genes and proteins in various iron-overload disorders in animal models and in humans [9].

## 2. Systemic and Brain Iron Metabolism

### 2.1. Absorption

A crucial event in iron homeostasis is absorption through diet. A dysregulation of dietary iron absorption leads to iron overload or iron deficiency. Between 1 and 2 mg of dietary iron is absorbed in the duodenum per day, and this is counterbalanced with losses from sloughing of mucosal and skin cells, menstruation, and other blood losses [9].

In humans, body iron varies between 3 and 5 g and most of it (~80%) is distributed between hemoglobin from erythrocytes and developing erythroid cells. Significant amounts of iron are also found in myoglobin, and the excess of the metal is stored in the liver as well as the macrophages of the reticuloendothelial system [10].

There are two forms of dietary iron: the heme (a prosthetic group of several enzymes composed of protoporphyrin IX and a Fe^2+^ ion) and the nonheme (ionic) forms (2/3 heme iron, 1/3 inorganic iron), both of which are absorbed at the apical surface of duodenal enterocytes via different mechanisms. Dietary heme (derived from myoglobin from red meat or blood hemoglobin) is transported across the apical membrane by an incompletely characterized pathway [4, 11, 12]. Heme iron absorption has been shown to be modulated by transporters formerly called heme carrier protein 1 (HCP1). However, the main physiological function of these transporters is folate transport. For this reason, these proteins in charge of heme iron absorption are now named proton-coupled folate transporter/HCP1 [4]. Once internalized in the enterocyte, heme iron is metabolized by heme oxygenase-1 (HO-1) which liberates Fe^2+^. Nonheme iron exists mainly as Fe^3+^, a nonbioavailable form which requires reduction in the intestinal lumen by ferric reductases before it can be transported across the apical membrane of the enterocytes by the divalent metal transporter 1 (DMT1) [8, 13, 14]. No matter where Fe^2+^ comes from, that is, heme metabolization or nonheme reduction, it is either complexed with ferritin (storage) or transported across the basolateral membrane of the enterocyte into the bloodstream via the Fe^2+^ transporter ferroportin (FPN) [8]. During efflux, Fe^2+^ is oxidized again to Fe^3+^ by the ferroxidase hephaestin [10, 15] which is thought to work together with FPN. Evidence indicates that ceruloplasmin, a plasma ferroxidase known to share significant homology with hephaestin, also participates in iron export from enterocytes and its binding to transferrin as well [16]. Once exported, iron is transported as a redox nonreactive form by transferrin (Tf) to tissues [17–19].

### 2.2. Transport

At any given time, relatively little of the iron in the body is in transit. As stated above, the transport of iron occurs via the serum transport protein Tf. Serum Tf is a glycoprotein of 80 kDa able to bind two Fe^3+^ with high affinity, and, under physiological conditions, only 30% of the Tf is saturated with iron [20]. The Tf iron pool is highly dynamic: only a small part of it comes from diet-absorbed iron and most of it comes from continuously recycled iron from senescent red blood cells. Macrophages from the reticuloendothelial system metabolize heme from effete erythrocytes and release iron (Fe^2+^) to the bloodstream through FPN in a process that involves reoxidation to Fe^3+^ (catalyzed by ceruloplasmin) and binding to Tf.

Developing erythroid cells take Fe^3+^ from plasma Tf. Tf releases iron within the cell in a process that involves interaction of Tf with cell surface receptors (TfR), internalization of the Tf-Fe^3+^ complex in a vesicle (endosome), acidification of the endosome to pH 5.5 via a proton pump-mediated mechanism, release of Fe^3+^ from Tf in low pH (Tf remains bound to TfR), reduction of Fe^3+^ to Fe^2+^ by a ferrireductase (Steap3), transport of Fe^2+^ across the endosomal membrane by DMT1 to the cytosol, dissociation of the Tf-TfR complex (their affinity is drastically reduced after the release of iron), and secretion of the apo-Tf into the bloodstream to recapture Fe^3+^ [9].

In the particular case of the brain, iron uptake occurs through the blood-brain barrier (BBB). Tf-Fe^3+^ complex is picked up through TfR located at the surface of the cerebral capillary endothelium [21]. Neurons also express TfR, and a homolog Tf receptor named TfR2 is known to be expressed in dopaminergic neurons. Among the non-Tf bound iron uptake, it has been proposed to be incorporated in neurons by the proton-driven transporter DMT1 or another iron transporter [22]. However, under physiological conditions, this is still a matter of debate [23]. Upon uptake, FPN and DMT1 mediate the export of iron from endothelial abluminal membranes of BBB to the cerebral compartments [24]. In brain interstitial fluids, iron is bound to Tf and becomes available for neurons and neuroglia that express TfR [25].

It is worth mentioning that iron concentration varies in different areas of the brain according to the levels of TfR expression. This pattern is due to the uneven distribution of TfR in the cerebral endothelia. For example, the hippocampus and the striatum present the highest metal concentration, whereas the cortex and the brain stem contain the lowest iron levels [26].

Lactoferrin is another iron-binding protein involved in brain iron uptake via a specific receptor (lactoferrin receptor, LfR). LfR has been demonstrated to be expressed in blood vessels and nigral dopaminergic neurons [27], suggesting that this receptor may be related to iron incorporation in the brain [28]. Moreover, an increase in LfR in dopaminergic neurons has been reported in patients with Parkinson's disease (PD) compared to control subjects. Interestingly, the greater LfR expression, the higher dopaminergic neuron degeneration. Thus, the increase in LfR has been associated with iron accumulation in vulnerable neuronal populations [27, 29]. However, the exact mechanism by which iron is augmented in the brain of PD patients still remains unclear.

### 2.3. Storage

The main storage organ for iron is the liver. Hepatocytes accumulate this metal as ferritin or hemosiderin. Ferritin is composed of a protein coat and an iron core of hydrous ferric oxide containing variable amounts of phosphate [30]. Around 4,500 iron atoms can be reversibly stored within the protein coat in a soluble complex, thus preventing iron toxicity by sequestering it in a nontoxic form. Hemosiderin stores iron in a form very similar to that in ferritin, but the iron-protein complex is insoluble.

In the central nervous system, iron excretion is extremely low [31]. Until aging, the presence of iron in neurons is almost exclusively limited to its ferrous form [32, 33]. With increasing age, neurons from different brain areas raise their content of iron and ferritin. However, ferritin content in neurons differs from that observed in oligodendrocytes, suggesting that neurons may have particular mechanisms for iron handling [34].

Mitochondrial ferritin (FtMt) is another iron storage protein. The amino acid sequence of FtMt shares high homology with H-chain ferritin indicating similar functions for both proteins [35]. It has been shown that FtMt expression is limited to tissues with high metabolic activity and oxygen consumption, among them, brain, testis, and heart [36, 37].

Neuromelanin, a dark pigment present in catecholaminergic neurons, is another iron-binding protein [38]. The exact role of neuromelanin in brain iron metabolism is still unknown, but its interaction with iron has been extensively studied in the parkinsonian *substantia nigra* [38–40]. As ferritin is mainly located in glia rather than neurons, it is possible that neuromelanin could regulate neuronal iron levels. Iron binding by neuromelanin may upregulate free radical formation [41] or may act as a hydroxyl radical scavenger [42, 43]. A recent proteomic study of neuromelanin isolated from human *substantia nigra* confirms its role in the homeostasis of neuronal iron [44]. In addition, diminished neuromelanin content in PD patients supports the function of this pigment in iron binding and the regulation of oxidative stress as well.

### 2.4. Regulation of Systemic and Brain Iron

Hepcidin, a liver-synthesized hormone, regulates the ferroportin-mediated transport of iron from enterocytes and macrophages to the bloodstream [45]. Hepcidin is able to bind to FPN and induce its phosphorylation and lysosomal degradation [30, 46]. Iron intake results in hepcidin accumulation with decreased iron absorption from diet. On the contrary, iron deficiency states promote a decrease in hepcidin levels, which stimulates duodenal iron absorption. Also, inflammatory states foster hepcidin accumulation and iron retention in macrophages. As hepcidin regulation is a complex mechanism, the discussion on the large number of proteins and transcription factors involved in this process goes beyond the aim of this review. It is worthy to note, however, that although the regulation of iron efflux by hepcidin is of great importance, the expression of FPN is also subjected to transcriptional and posttranscriptional control [10].

Several studies have shed light on the expression of hepcidin in different brain areas such as the cortex, the hippocampus, and the spinal cord [47, 48]. Both neurons and glial cells have been shown to express hepcidin in these brain regions. An increase in hepcidin levels has been demonstrated in the choroid plexus during inflammatory processes. Reinforcing the regulatory role of hepcidin in brain iron metabolism, it has been demonstrated that overexpression of this protein decreases FPN levels and provokes iron overload and, in consequence, neurodegeneration [47].

## 3. Iron and Oxidative Stress

In cells, iron concentration ranges between 0.2 and 1.5 *μ*M and is weakly bound to low-molecular weight substrates. The ability of iron to change between 2+ and 3+ valency states with a redox potential compatible with the cellular environment renders it one of the most important metals in catalytic processes of oxidative biology [49, 50]. As stated above, the appearance of oxygen in the atmosphere made it possible for the organisms that adapted to those conditions to get 20 times more energy than that obtained from fermentative reactions [51]. However, the negative part of this issue is the continuous generation of reactive oxygen species (ROS) as normal by-products of metabolism. The redox equilibrium is essential for the physiology of the body, and since ROS first appeared, they have been involved in the regulatory mechanisms of synthesis and homeostasis of biomolecules, as well as many important processes of the organism [52]. Although small amounts of free radicals are produced in all metabolic processes (enzymatic reactions in the endoplasmic reticulum, microsomes, peroxisomes, or cytoplasm), the major generation of ROS emerges by incomplete reduction of dioxygen in the mitochondrial electron transport chain. In brief, the first radical formed from O_2_ is the superoxide radical (O_2_^•^), which is not itself very reactive but it is able to generate other dangerous species. The addition of a second electron to a superoxide radical results in the peroxide ion (O_2_^2−^). Peroxide is not a radical, and when protonated, it generates hydrogen peroxide (H_2_O_2_). The following reduction of hydrogen peroxide (through the metal-ion catalyzed Fenton reaction) produces the hydroxyl radical (OH^•^) which reacts rapidly with high affinity with almost every molecule found in living cells [51, 53–55]. Under normal conditions, ROS are rapidly detoxified by the cell, but under certain circumstances in which ROS production exceeds intracellular antioxidant defense, an increase in the steady state concentration of ROS is observed: a condition known as oxidative stress.

*In vitro* experiments have shown that cellular oxidative stress induced by iron overload is characterized by increased lipid peroxidation and protein and nucleic acid modifications [56–59]. The presence of a labile iron pool (LIP, redox-active iron bound to low affinity compounds and which determines the iron status of the cell) is the main contributor to oxidative stress during iron overload [60]. This destructive potential of iron has led to investigate its role in the pathophysiology of several neurodegenerative diseases associated to oxidative stress, and this is the main focus of this review.

## 4. Evidence of Iron Accumulation in the Brain

The increased human lifespan of today has had a significant impact on the development of neurodegenerative diseases in elderly people. Metalloneurobiology, a relatively new discipline, has become extremely important for establishing the role of transition metals in neuronal degeneration. Iron is required for usual metabolic processes, such as mitochondrial respiration and DNA synthesis, and it also plays a key role in the biosynthesis of neurotransmitters and myelin in the brain [51, 61]. Moreover, iron has been demonstrated to be necessary for the normal development of cognitive functions. In this regard, iron deficiency early in life has shown to cause learning and memory impairment in humans [62–64]. Additionally, it has been observed that this metal ion progressively accumulates in the brain during normal aging [65]. However, this accumulation has also been related to the pathogenesis of several neurodegenerative disorders, such as PD and Alzheimer's disease (AD) [61]. In this connection, interesting studies have been conducted on *Octodon degus*. This Chilean rodent, widely utilized for modeling sporadic AD, has shown increased levels of redox-active metals (Fe, Cu, and Zn), specifically in the cortex and the hippocampus, the brain areas mainly affected in AD [66]. Indeed, increased iron in the *substantia nigra* of PD patients has been related to neurodegenerative mechanisms and, notably, clinical studies using iron chelators have shown to lower iron levels and improve the performance of early diagnosed PD patients [67].

In the abovementioned disorders, iron-induced oxidative stress, combined with defective antioxidant capacities, promotes neuronal death and neurodegeneration [4, 22, 68]. However, it is still uncertain whether the extensive brain iron accumulation is a primary cause of the pathogenic event, or just a consequence of a previous dysfunction [69, 70]. Interestingly, the diseases collectively known as neurodegeneration with brain iron accumulation (NBIA), all of which are characterized by iron accumulation in basal ganglia and mutations in proteins involved in iron traffic or metabolism, have shown clinical and molecular similarities with neurodegenerative diseases such as AD and PD [71–73]. Importantly, iron deposition has been reported to occur only in specific brain regions in patients with chronic neurodegenerative diseases [4, 68, 69, 74]. In the case of AD, iron accumulates mainly in the cerebral cortex and hippocampus, without a concomitant increase in ferritin normally observed in aging [74], thereby raising the risk of oxidative stress. Both neurodegenerative diseases present neuroinflammation as a triggering factor for neuronal death. A recent study reports that the inflammatory process related to neurodegeneration causes an increase in iron levels and ferritin in microglia and neurons as well [75]. Moreover, the release of iron from dying neurons, glial cells, and macrophages to the extracellular space has been postulated as a source of iron to be taken up by nonaffected neurons in a TfR-mediated pathway [75]. Tf-independent iron uptake has also been described. This pathway is known to induce ferritin expression to serve as an iron scavenger [76]. A balance between the expression levels of ferritin and ferroportin (which mediates neuronal iron excretion) has been reported in neurons that usually deal with large amounts of iron [75]. However, although there is strong evidence of a link between iron and neurodegeneration, further studies that correlate the temporal relationship need to be carried out. Most importantly and although in its infancy, the development of new living models makes it possible to observe the detailed and unambiguous molecular events occurring in the degenerative process urges.

## 5. Intersections between A*β* and Iron in the Brain: Iron Chelation Therapies

It is well established that the pathological hallmarks of AD in the brain include abundant extracellular amyloid *β* peptide (A*β*) plaques, intracellular neurofibrillary tangles of protein tau, and increased brain iron (in and around A*β* plaques) [77, 78]. In this review, we focus particularly on A*β* and iron interactions. The aggregation of A*β* (which has been shown to be toxic to neurons) is known to be triggered by metals such as zinc, copper, and iron [79–82]. Therefore, metal dyshomeostasis may be an important factor leading to AD pathogenesis. Moreover, A*β* has been demonstrated to bind zinc and iron, and more strongly, copper [83]. Therefore, these chelation characteristics of the peptide may account for the enrichment of these metal ions in A*β* plaques [83].

Cumulative evidence suggests that A*β* is the major cause of neurotoxicity and may significantly contribute to synaptic dysfunction in AD [84]. Iron accumulation in affected brain regions, as reported by *postmortem* and magnetic resonance imaging (MRI) studies [85–87], may also be responsible for the increased oxidative stress observed in AD [50, 88]. A*β* has broadly been shown to bind iron [89]. This A*β*-iron interaction is through His6, His13, and His14 of A*β* and is thought to be facilitated in a more reduced environment such as the brain due to the prevalence for the ferrous form of iron to bind A*β* [50, 90]. Interestingly, a very recent study carried out on APP/PS1 mice has demonstrated that ferrous iron is an integral part of amyloid plaques and has provided evidence that (supporting previous *in vitro* studies) A*β*-induced reduction of iron is able to occur *in vivo* [91]. On the other hand, ROS generated through iron-aggregated A*β* are toxic to neurons [92] and would partially contribute to the neurotoxicity present within the iron-enriched environment around senile plaques [93, 94]. Moreover, accumulated iron in neurofibrillary tangle-containing neurons and the neuritic processes adjoining senile plaques in AD [95] have been correlated with cognitive decline [96].

**Figure 1:**
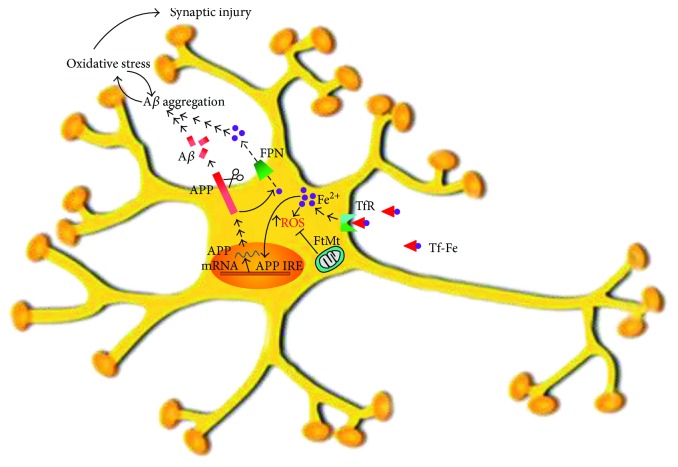
Iron and A*β* interactions. Transferrin-bound iron is taken up by the neuron via a receptor- (TfR) mediated mechanism. An augmented pool of intracellular iron (mainly as Fe^2+^) increases ROS production with the concomitant generation of oxidative stress. Mitochondrial ferritin is able to protect the neuron against iron-induced oxidative stress. Iron itself is able to induce APP expression through an APP IRE. Also, APP mediates iron export via FPN. An increased APP expression (due to an increased iron uptake) results in an increased A*β* generation. Interestingly, iron is involved in A*β* aggregation in a mechanism that generates oxidative stress, but it is also known that previous oxidative stress increases A*β* aggregation. In this way, both A*β* and iron participate in a vicious cycle known to culminate with synaptic oxidative injury.

As iron homeostasis is so important in preventing cell oxidative damage, mechanisms to keep iron in the physiological concentration range have been evolutionarily incorporated to maintain optimal cell function [10]. The proteins required to regulate cellular iron homeostasis in the brain are quite the same as those used in the body's periphery: the iron response proteins (IRPs) 1 and 2 bind to their respective iron regulatory elements (IREs) in either the 3′-untranslated region (UTR) or 5′-UTR of an mRNA. Despite studies suggesting that iron nonspecifically coprecipitate with A*β* in AD [50, 97], a role for iron in A*β* metabolism and AD progression has been shown and, conversely, A*β* seems to be involved in neuronal iron homeostasis. Not only has iron been demonstrated to bind to and accelerate A*β* precipitation [90, 98] but it also regulates its generation from amyloid precursor protein (APP) (Figure 1). The aforementioned A*β* plaques are mainly constituted by a 40–42-amino acid A*β* peptide cleaved from the APP by *β*- and *γ*-secretases in the amyloidogenic pathway [83, 99]. Interestingly, intracellular iron levels have been reported to control APP translation via an IRE RNA stem loop in the 5′-UTR of the APP transcript. This APP IRE has been found to physiologically bind with IRP1, and not with IRP2 in human neural cells [100]. Therefore, increased cytosolic iron levels translationally upregulate APP expression [99, 100]. Interestingly, APP has been found to facilitate neuronal iron efflux through a mechanism that involves FPN [101, 102] (Figure 1). Hence, iron influx drives the translational expression of the neuronal APP, which, in turn, has a role in iron efflux. In this regard, intracellular iron retention has been reported in neuronal cultures and mouse models depleted of APP, and normal iron efflux has been observed to be restored when APP was either added extracellularly [101] or overexpressed [101, 103]. It is also worth noting that Down's syndrome-suffering children (who have increased APP expression) have been found to present anemia and a high risk of iron deficiency [50, 104, 105].

Another piece of evidence between the link of iron and A*β* comes from the recently described neuroprotective role of FtMt. In neurons exposed to A*β*25-35, the overexpression of FtMt diminished the labile iron pool, decreased oxidative injury, and prevented cytochrome c release from mitochondria through the activation of the mitogen-activated protein kinase (MAPK) pathway, thus inhibiting neuronal apoptosis [106]. Moreover, an increased FtMt expression has been described in AD [107, 108].

In view of the crucial and multifactorial role of iron in AD progression, it is imperious to consider this metal in the therapeutic design. Metal chelation is one of the therapeutic strategies for AD [109]. The great challenge will be developing chelating drugs able to cross the BBB. It has been demonstrated that A*β*-insoluble aggregates are dissolved by metal chelators [110]. Moreover, the iron chelator desferrioxamine has shown to decelerate disease progression [111]. However, due to the size of desferrioxamine and the relative impermeability of the BBB to this drug, desferrioxamine has had limited success in the brain when administered peripherally [112]. Fortunately, smaller molecular compounds with chemical modifications have had promising results in preclinical trials [113, 114]. For instance, deferiprone has been approved for peripheral iron overload in thalassemia and in the neurodegenerative disease, Friedrich's ataxia [115]. Additionally, clioquinol derivatives have been used in clinical trials in AD patients, showing reduced A*β* in cerebrospinal fluid, as well as improved cognitive performance [116]. A key point to take into account in the chelation therapy would be to restrain iron without generating a deficiency which would be as dangerous as the excess of the metal.

## 6. Signaling of Iron in the Central Nervous System

The activation or inactivation of specific signaling pathways is one of the multiple responses a neuron can give to oxidative stressors. The decision of living or dying in a certain cell type will depend on several aspects of the stress (type, extent, and time of exposure) which will determine the signaling pathways that will result in being turned on and off. The sum of these aspects will define the cellular fate [117, 118]. In this respect, iron has been involved in the triggering of several protective and proapoptotic signaling pathways in neurons [119].

It has been demonstrated that synaptic endings are the sites where the first signs of neurodegenerative processes are likely to appear. It is well known that marked synapse loss, rather than neuronal death, occurs during the initial events of AD [119–121]. This fact might involve apoptotic cascades triggered locally at the synapse that might occur independently of gene transcription. It has been reported that morphological changes in the synapses precede A*β* deposition [122]. Also, a decrease in synaptic markers has been observed in hippocampal neurons exposed to Fe^2+^ [57]. Synaptic susceptibility to iron-induced oxidative stress has been largely shown [123–129], being that synaptic endings from senile animals are more vulnerable to Fe^2+^ exposure than those isolated from adult animals [128]. Increased membrane lipid peroxidation, loss of selective permeability of plasma membrane, 4-hydroxynonenal (HNE) generation, impairment of membrane ion-motive ATPases, glucose and glutamate transport, and mitochondrial function have been reported to be constant observations of synaptosomal oxidative injury induced by iron exposure [123–129].

Regarding synaptic iron-triggered signaling, phosphatidylinositol 3-kinase (PI3K)/Akt pathway, a well-known survival-associated pathway, has been reported to be activated in synaptic endings of both adult and aged animals; the difference has been found mainly in the time frame of the activation, which varies with animal age [128]. One of the best known Akt substrates, glycogen synthase kinase 3*β* (GSK3*β*), has been largely reported to be involved in metabolism, survival, gene expression, and cytoskeletal dynamics, and it is considered a crucial player for determining neuronal fate [130]. The phosphorylation (and inactivation) of synaptic GSK3*β* has been shown to follow the same time pattern as Akt [128]. Extracellular signal-regulated kinases (ERK) 1/2, key components of stress-related cellular responses that have been involved in both survival and death, have been found to present different patterns of synaptic activation according to the age of animals. Moreover, the dependence of ERK1/2 activation on the PI3K pathway has also shown to be age-dependent [128].

Kuperstein and Yavin have described the effect of iron and A*β* on ERK signaling in neuronal cultures [131]. Interestingly, a two-peaked activation of ERK has been shown: a rapid one (5 min) followed by a decline by 30 min and a second one (continuous up to 24 h) in which nuclear translocation of ERK is detected. Desferrioxamine, as well as antioxidant treatments, has shown to suppress ERK activation and nuclear translocation, resulting in a reduced apoptotic death. In the light of these observations, it can be concluded that ERK promotes iron-triggered damage. In extending these findings, it has also been demonstrated that the coexposure of neuronal cells to iron and A*β* induces a decrease in Akt activity and Bad phosphorylation, and an increase in the activation of p38 and caspase-3 and caspase-9 [132]. It is important to highlight that there are many controversies about the effect of iron and A*β* (each alone or combined) on cellular signaling cascades. Differences may be due to the specific experimental conditions (time of exposure, concentration of the metal or A*β*, and cellular type). The signaling lipid phosphatidic acid, produced by phospholipase D (PLD), has been also involved in the synaptic response induced by iron overload. Moreover, PLD1 and PLD2 activities have shown to be involved in the activation of protein kinase D (PKD) 1, ERK1/2, and protein kinase C (PKC) *α*/*β*II in adult animals, but not in senile animals [133]. These molecular differences in the signaling reported in aged animals could account for the increased age-related synaptic susceptibility to iron and might also suggest that these signaling cascades pursue different goals in the synapses according to age. Indeed, PLD2 has been shown to be activated by oligomeric A*β* in cultured neurons [134]. Moreover, A*β* has been proven to fail in the suppression of long-term potentiation in PLD2-deficient mice hippocampal slices, suggesting that PLD2 is required for the synaptotoxic action of A*β* [134]. Experiments carried out in a transgenic mouse model of AD (SwAPP) have also confirmed that PLD2 activity is increased and the ablation of its gene (*Pld2*) ameliorates memory deficits and protects the synapses [134]. These findings highlight the capital role of PLD in AD progression.

As mentioned, iron (alone or in combination with A*β*) is able to activate different signaling cascades. These signaling pathways are the molecular events necessary for the activation of several transcription factors which, by regulating the expression of specific genes, modulate the neuronal response to the injury. Extensive evidence links the transcription factors Forkhead box O (FoxO), nuclear factor- (NF-) *κ*B, and activator protein (AP) 1 to neuronal responses to oxidative stress [57, 135]. FoxO transcription factors have been shown to increase stress resistance and to extend lifespan in *Drosophila* [136]. Recent studies have demonstrated that phosphorylated FoxO3A translocates out of the nucleus in HT22 neurons in a PI3K/Akt-mediated mechanism after iron exposure [57]. Surprisingly, this defensive response to oxidative stress leads to downregulation of superoxide dismutase (SOD) 1 and 2 expression, so that the neuron responds to the oxidative injury via glutathione metabolism [57]. Interestingly, the neuron has been shown to respond to oligomeric A*β* exposure with the same insulinomimetic signaling as it does upon iron exposure, although undetectable oxidative stress markers have been reported in the presence of A*β* [137]. NF-*κ*B plays crucial roles in cellular resistance to oxidants and survival. The role of this transcription factor in the inhibition of apoptosis (by participating in the induction of antiapoptotic genes) has been well demonstrated [138]. As to iron and AD, NF-*κ*B has been associated with increased resistance of neurons to apoptosis induced by exposure to the metal ion [135]. The expression of this transcription factor has been found to be increased both in neurons and astrocytes in areas adjoining A*β* plaques in patients with AD [139]. There is also evidence about A*β*-induced activation of NF-*κ*B in cultured neurons, and both protective and injurious roles of this transcription factor have been reported [140–142]. For its part, AP-1, an essential transcription factor in cellular response to oxidative stress (as well as in other processes such as proliferation, differentiation, and survival), c-Jun-N-terminal kinase, and p38 (two stress-related MAPK which are upstream of AP-1 in the cascade) have also shown to be activated by oxidative stress [135]. Both MAPK, as well as AP-1, have been shown to be involved in physiological functions of the brain. Interestingly, c-Jun, a component of AP-1, has been recently attributed a dual role: it is able to mediate plasticity and repair mechanisms, but it is also thought to participate in neuronal death [143].

The role of free calcium in neuronal death induced by oxidative stress has been well documented [128, 144, 145]. Under physiological conditions, the intracellular calcium level is tightly controlled, and relatively small fluctuations in intracellular calcium concentration might cause neuronal deterioration and eventually lead to cell degeneration. Neuronal activity generates calcium signals that result in the transcription of genes that are crucial for synaptic plasticity and neuronal survival. It has also been demonstrated that calcium participates in the early events of iron-induced oxidative injury in synapses, but after long-term exposure to iron, the absence, as much as the excess of calcium, appears to be more deleterious to the synaptic endings than the damage induced by iron itself [128]. The exact mechanism by which calcium participates in synaptic injury is not clear. It seems that the activation of distinct signaling cascades downstream from key points of calcium entry at synapses has a major role in the neurodegenerative process. In the whole neuron, calcium released from intracellular stores has been linked to the stimulation of ERK and calcium-calmodulin-dependent protein kinases and cAMP response element-binding protein- (CREB-) dependent gene transcription [64]. The latter, a process known to be involved in synaptic plasticity, occurs through the calcium-induced calcium release, a compelling mechanism based on the calcium-dependent activation of calcium release channels, such as the ryanodine receptors (RyR) or the IP3 receptors (IP3R), and by which small and localized calcium signals can be amplified or propagated to the nucleus (also to the mitochondria) [64]. Interestingly, RyR activity has been shown to be redox sensitive, due to a few cysteine residues of the RyR protein [64, 146, 147]. For this reason, RyR, which have been proposed as redox sensors [64, 147], have attracted increasing attention in the research field of synaptic plasticity in hippocampal neurons [148, 149]. Work from Muñoz and colleagues has demonstrated that iron exposure of PC12 cells or hippocampal neurons leads to ROS generation and ERK activation through RyR-mediated calcium release. Both effects are clearly reduced by either mannitol (hydroxyl radical scavenger) or desferrioxamine (an iron chelator), indicating that iron-induced hydroxyl radicals are responsible for calcium release and ERK stimulation [150]. This is clear evidence of calcium and iron involvement in the events necessary for synaptic plasticity and supports the idea that increased iron concentration may cause neurodegeneration via excessive intracellular calcium [64].

## 7. Concluding Remarks

In this review, we summarize cutting-edge knowledge about iron and its involvement in neurodegenerative processes, in particular, AD. Vast evidence has demonstrated that iron is a clear generator of oxidative stress. Both iron and oxidative stress have been linked to A*β* aggregation. However, the exact order in the molecular events that lead to the onset of AD still remains elusive. Exploring the brain's own iron homeostatic mechanisms and the signaling events involved in response to metal overload may shed light on several unclear aspects of the disease and may therefore lead straightaway to the development of a definite therapeutic tool.

## Abbreviations

HNE: 4-Hydroxynonenal
AD: Alzheimer's disease
APP: Amyloid precursor protein
BBB: Blood-brain barrier
DMT1: Divalent metal transporter 1
ERK: Extracellular signal-regulated kinases
FPN: Ferroportin
GSK3*β*: Glycogen synthase kinase
HCP1: Heme carrier protein 1
HO-1: Heme oxygenase-1
IRE: Iron regulatory elements
IRP: Iron response protein
LIP: Labile pool iron
LfR: Lactoferrin receptor
MRI: Magnetic resonance imaging
FtMt: Mitochondrial ferritin
NBIA: Neurodegeneration with brain iron accumulation
PD: Parkinson's disease
ROS: Reactive oxygen species
TfR: Transferrin receptor
Tf: Transferrin
UTR: Untranslated mRNA.
